# Musings from a Vectosaur: Malaria in 2026

**DOI:** 10.5281/zenodo.18923229

**Published:** 2026-03-10

**Authors:** Manuel F. Lluberas

**Affiliations:** 1 HC1 Box 13526, Río Grande, Puerto Rico, 00745, USA.

## Abstract

For more than two decades, global malaria reports have presented an encouraging narrative: expanding programme coverage, billions invested, and steady declines in mortality. Yet those who spend their evenings beside light traps and breeding sites often see a more complicated reality. Beneath the reassuring graphs lies a persistent operational question: Are we winning the fight against malaria, or are we simply becoming better at reporting progress while our ability to measure it remains uncertain? The 2025 Africa Malaria Progress Report captured this tension candidly, noting that “behind the figures lies a stark truth: we remain off-course and the ‘perfect storm’ of threats has intensified.”

I argue that the problem confronting malaria control today is not merely financial or clinical. Rather, it is structural. Modern malaria programmes rely overwhelmingly on two tools: Long-lasting insecticidal nets and indoor residual spraying. Both interventions have saved countless lives, but neither was ever designed to carry the entire burden of vector control indefinitely. Under sustained pressure, mosquito populations are responding exactly as evolutionary biology predicts: Through increasing insecticide resistance and shifts in feeding and resting behaviour that circumvent indoor interventions. At the same time, new ecological challenges, including climate change and the expansion of *Anopheles stephensi* into African urban environments, are adding complexity to already strained systems. Equally troubling is the fragmentation of malaria control across numerous institutions, funding streams, and implementation structures that often operate in parallel rather than as a unified system. Re-establishing integrated vector management by combining surveillance, larval source management, environmental control, and targeted adult mosquito control interventions under coordinated national programmes may offer a more resilient path forward. Mosquitoes respond to pressure. Successful malaria programmes must learn to do the same.

Nobody asked me, but after a lifetime chasing mosquitoes across continents, a few things still keep me awake at night.

Spend enough evenings leaning against a mud hut or dusty vehicle while a light trap hums in the background and you start noticing patterns, both in mosquito behaviour and in human institutions. The mosquitoes, oddly enough, are usually the more predictable of the two.

Over the years I have learned to be suspicious of tidy narratives. Malaria control, unfortunately, has developed a fondness for them.

If you read the global reports from the past two decades, you might conclude that humanity has been steadily marching toward victory. Mortality down. Coverage up. Billions spent. Programmes expanded. Graphs pointing reassuringly downward [1]. And yet, anyone who has spent time in the field knows that the story is rarely that simple.

Years ago, while reading a global malaria report published by WHO that proudly announced a dramatic reduction in mortality, I stumbled across a line buried deep in the technical sections. It explained that in a large share of the countries reporting, places carrying most of the malaria burden, it was “not actually possible to reliably assess trends” because “surveillance systems were weak and unreliable.”

That short statement stopped me cold. So, which was it? Are we winning, or are we unable to measure the score?

That question has never quite gone away.

Fast forward to today and the language has become even more revealing. The 2025 Africa Malaria Progress Report [2] includes a sentence that deserves to be read slowly:

*“Behind the figures lies a stark truth: we remain off-course and the ‘perfect storm’ of threats has intensified.”* (Page i)

For those of us who have spent our careers in mosquito control, that sentence feels less like a revelation and more like confirmation of something we have been quietly observing for years. The storm didn’t suddenly appear. We have been sailing toward it with all sails up for quite some time.

Anyone who has worked in malaria control long enough knows a simple truth: our operational toolbox is not very large. At the moment, vector control efforts rely overwhelmingly on two strategies, long-lasting insecticidal nets (LLINs) and, to a lesser extent, indoor residual spraying (IRS). Both have their place. Both have saved lives. But they were never meant to carry the entire burden of malaria control alone.

In earlier decades, vector control programmes attacked mosquitoes from multiple directions simultaneously. Environmental management, larval source management (LSM), targeted spraying, housing improvements, surveillance, and rapid response teams all played a role. Programmes were built as integrated systems rather than as single-tool campaigns. Somewhere along the way, that integrated approach began to erode.

Today, LLINs have become the centrepiece of malaria policy to such an extent that suggesting the implementation and expansion of other tools can sometimes feel like proposing heresy. I have sat in any number of international meetings where the spoken logic has been that “every dollar spent on vector control beyond nets is somehow a dollar taken away from the solution.”

Field entomologists tend to view the matter differently. Mosquitoes are not policy documents. They are adaptable biological systems that respond to pressure. Apply a single intervention across vast areas year after year and the vector will respond in the only way evolution allows: adapting.

Which brings us to the first gusts of that “perfect storm.”

Insecticide resistance is no longer an emerging problem. It is an established reality in many regions. Pyrethroids, the backbone of net treatments and IRS for decades, face growing resistance across large portions of African vectors. And historical tools like DDT remain politically contentious even when operationally effective.

But resistance is only part of the story. Mosquitoes are not just developing chemical defences. In many areas they appear to be modifying their behaviour as well. Increased outdoor feeding, shifts in biting times, and changes in resting patterns have been reported in multiple regions where indoor-focused interventions dominate. For a vector control specialist, these observations are not particularly surprising. They are exactly what evolutionary pressure would predict.

And then, as if the situation needed further complexity, *Anopheles stephensi*, a container breeder historically associated with urban malaria transmission in Asia arrived in Africa as if on queue. Its continued territorial expansion introduces new ecological and operational dynamics into cities that were not previously considered high-risk environments.

If one were designing a “perfect storm” scenario for malaria control, these ingredients would certainly make the list. Yet our operational adjustments remain remarkably absent.

Another puzzle that has long fascinated and occasionally frustrated those of us working in the trenches is the remarkable fragmentation of malaria programmes. Consider the number of organisations involved: international agencies, bilateral initiatives, philanthropic foundations, development banks, governmental and non-governmental agencies and organisations, national programmes (NM-CPs or NMEPs), academic partners, and private contractors. Each operating with its own funding streams, reporting structures, timelines, and priorities.

Individually, many of these efforts are impressive. Collectively, they resemble a flotilla racing sailboats each trying to get to the finish line first to get a trophy.

It raises a simple question that “old-timer” entomologists occasionally whisper to each other in conference hallways:

**Why has there never been a true global coordination forum where the major malaria actors sit together, not for speeches or declarations, but for meaningful operational planning?**

Imagine bringing together the technical teams responsible for pesticide evaluation, malaria programme strategy, major financing mechanisms, philanthropic investments, malaria drugs and vaccines, and operational implementation. Not as a ceremonial event but as a working session focused on one task: designing a genuinely integrated malaria control strategy.

Such a meeting would not be glamorous. It would involve spreadsheets, operational maps, cross-border collaborations, surveillance data, and long discussions about logistics. But it might produce something palpably absent: a unified strategy deploying every available vector control tool in a coordinated way and tailored to the vector and local conditions.

Instead, what we often see are parallel programmes visiting the same communities for different interventions. One team distributes mosquito nets. Another conducts limited indoor spraying. A third might occasionally discuss larval source management with residents. Each programme reports success within its own metrics, but rarely as a single year-round regional or national system.

From a programme management perspective, this inefficiency is striking. At least three separate programmes, budgets, logistical chains, sets of staff rotations, and administrative structures often serving the same villages or regions. Anyone responsible for running a national vector control service should immediately recognise the opportunity for consolidation.

Larval source management provides a good illustration. In many modern malaria programmes, it is treated as a supplementary intervention: useful perhaps in urban settings but rarely prioritised at scale. Yet historically, controlling larval habitats was a central pillar of mosquito management and responsible for the control or eradication of mosquito-borne diseases.

The reason is simple: mosquitoes are easier to control as larvae when they are concentrated in water than as adults flying across the landscape looking for blood meals. Of course, larval control is not universally applicable. Geography matters. Hydrology matters. Infrastructure matters. But in places where it can work, ignoring it entirely seems less like strategy and more like habit.

The same could be said for environmental management, housing improvements, and localised vector suppression techniques. None of these approaches replaces nets or spraying. But together, they create multiple pressure fronts on the vector population.

Mosquitoes, like sailors facing shifting winds, struggle when forces come from several directions at once.

The financial scale of modern malaria programmes is gigantic. Tens of billions of US Dollars have been invested globally over the past two decades. No one disputes that the investment has saved many lives. But when a system continues to rely on essentially the same limited strategy year after year, while the vector adapts, new ones invade, and the disease persists, it’s fair to ask whether the allocation of those resources is optimal.

Taxpayers in donor countries often assume that malaria programmes operate with the same integrated discipline seen in successful eradication campaigns of the past. Unfortunately, field practitioners know the reality is more complicated.

The African Leaders Malaria Alliance 2025 African Malaria Progress Report and others warning that we are “off-course” is acknowledging something many of us have sensed for some time: Progress has slowed, risks are increasing, and the strategy requires recalibration.

Nobody asked me, but my suggestion would be fairly straightforward. Treat malaria vector control as what it truly is: An operational system, not a collection of separate projects, or a clinical condition. Build permanent national vector control services rather than temporary clinical campaigns. Train and fund professional field teams who remain in place year-round. Integrate entomological and epidemiological surveillance, larval control and LSM, mosquito net distribution, IRS, and community engagement under a unified management. Most importantly, deploy multiple tools simultaneously rather than sequentially. Mosquitoes respond to pressure. Programmes should do the same.

Will that approach eliminate malaria overnight? Of course not. But it would move us away from the peculiar situation we find ourselves in: Repeating the same strategy annually while expressing surprise that the outcome changes only marginally. Which brings to mind a line often attributed to Einstein about insanity and repetition.

The people working in malaria control are intelligent, committed, and deeply motivated by the desire to save lives. But institutions, like ecosystems, sometimes drift into stable patterns that become difficult to change.

Perhaps the growing recognition that we are “off-course” will provide the necessary push. Until then, some of us old vectosaurs will keep asking the inconvenient question: *Are we doing the right thing? Are we doing things right?*
